# The impact of varicella vaccination on paediatric herpes zoster epidemiology: a Canadian population-based retrospective cohort study

**DOI:** 10.1007/s10096-021-04298-z

**Published:** 2021-06-26

**Authors:** Ellen Rafferty, Laura Reifferscheid, Margaret L. Russell, Stephanie Booth, Lawrence W. Svenson, Shannon E. MacDonald

**Affiliations:** 1 Institute of Health Economics, Edmonton, Alberta Canada; 2 Faculty of Nursing, University of Alberta, Edmonton, Alberta Canada; 3 Department of Community Health Sciences, Cumming School of Medicine, University of Calgary, Calgary, Alberta Canada; 4 Analytics & Performance Reporting Branch, Alberta Health, Government of Alberta, Edmonton, Alberta Canada; 5 Division of Preventive Medicine, University of Alberta, Edmonton, Alberta Canada; 6 School of Public Health, University of Alberta, Edmonton, Alberta Canada

**Keywords:** Herpes zoster, Varicella, Immunisation, Epidemiology, Chickenpox, Paediatric shingles

## Abstract

The impact of universal varicella vaccination on herpes zoster (HZ) risk in unvaccinated and vaccinated children, and its long-term influence on HZ epidemiology, remains unknown. We conducted a retrospective cohort study using population-based administrative health data for children born between 1993 and 2018 (n = 924,124). We calculated age-specific cumulative HZ incidence rates by vaccination status for cohorts born before (1993–1999) and after (2000–2018) programme implementation; results were used to calculate relative risk of HZ by age group, vaccination status and vaccine availability period. Annual HZ incidence rates were calculated for 1993–2018. HZ risk was higher among unvaccinated children compared to vaccinated children across age groups; 64% higher before universal vaccination (RR: 0.36, 95% CI: 0.33, 0.39), and 32% higher after universal vaccination (RR: 0.68, 95% CI: 0.64, 0.73). Among unvaccinated children, HZ risk was 60% lower after vaccine programme implementation (RR: 0.40, 95% CI: 0.38, 0.43). Two-dose receipt corresponded with a 41% lower risk of HZ compared to one-dose receipt (RR: 0.59, 95% CI: 0.53, 0.65). Crude annual HZ incidence rates declined 64% after programme implementation, with decreases observed across age groups. Universal varicella vaccination programme implementation corresponds to decreased paediatric HZ incidence across age groups, in both vaccinated and unvaccinated individuals. Results from this study can be used to help inform varicella vaccination programme decision-making in other countries.

## Introduction

Herpes zoster (HZ) results from reactivation of latent varicella zoster virus (VZV), introduced through primary varicella infection. Reactivation of VZV can occur decades after primary infection and is generally associated with a decline in cell-mediated immunity due to aging or immunosuppressive illness/medication. While most common among older adults, HZ does occur in healthy children and adolescents. Prior to varicella vaccination programmes, approximately 9% of HZ cases in Canada occurred in individuals less than 10 years of age [1, 2].

In Alberta, varicella vaccine was available through private purchase in 1999–2001, although coverage was less than 5% in 2001 [1], indicating minimal vaccine uptake via private purchase. Varicella vaccine was added to the publicly funded vaccination schedule in July 2001. The publicly funded programme offered universal vaccination at 12 months to all children born after 1999, while targeted catch-up programmes provided publicly funded vaccine for non-immune children and adolescents born after 1983 [1]. A second dose for children 4–6 years was added to the schedule in August 2012.

Varicella vaccination is effective at preventing primary varicella infection; research also indicates that vaccination can decrease individual risk of HZ [3–5]. However, as with wild-type VZV (wt-VZV), the live-attenuated VZV contained in the varicella vaccine (v-VZV) can establish a latent infection with potential to reactivate as HZ [5]. While studies consistently show the risk of HZ disease is lower in those vaccinated than those unvaccinated [4, 6] or with a history of varicella disease [3, 5], the longevity of this protection remains largely unknown. Uncertainties around the impact of varicella vaccination on HZ have led some developed countries to delay incorporating the vaccine in routine childhood vaccination programmes [7].

Universal varicella vaccination programmes can impact population level HZ rates, not only through increased individual vaccination coverage but also by decreasing the amount of varicella circulating in the community. Most studies have used ecological designs to evaluate the impact of universal vaccination programmes on paediatric HZ rates [8]. However, catch-up dosing programmes can complicate comparisons between “pre-vaccination” and “post-vaccination” era trends. Studies that have incorporated individual-level vaccination data show significant differences in HZ rates between vaccinated and unvaccinated children; however, these studies have been limited by small sample size and/or short post-vaccine follow-up periods and do not include data on HZ incidence rates prior to vaccine programme implementation [3–5, 9]. To date, there is little published evidence examining the impact of mature vaccination programmes on both vaccinated and unvaccinated children. In addition, no Canadian studies have examined the impact of individual vaccination status on population-level paediatric HZ risk [10]. This information is crucial for adequately assessing the health system impact of varicella vaccination programmes [11], and thus useful for informing decision-making in other countries considering implementing a varicella vaccination programme.

The objective of this study was to assess the impact of age and varicella vaccination status on medically attended paediatric HZ incidence rates. Two vaccine availability periods were of interest: the pre-universal vaccination era (birth cohort 1993–1999) and the publicly funded universal vaccination era (birth cohort 2000–2018). We sought to:

1. Estimate the relative risk of HZ among vaccinated children compared to unvaccinated children by age group and vaccine availability period.
2. Estimate the impact of a two-dose varicella vaccination series on HZ risk as compared to the one-dose programme.
3. Measure the annual incidence of paediatric HZ over the study period (1993–2018) and by age group.

## Methods

### Cohort and data sources

We created a retrospective, population-based cohort of all children born in Alberta between January 01, 1993, and December 31, 2018, using a birth registry (Vital Statistics Registry). All residents of Alberta are required to register with Alberta’s publicly funded, universal health care plan. Approximately 99% of Alberta residents are registered. Registrants are given a personal health number (a unique lifetime identifier). This identifier can be used to deterministically link administrative databases available through the Alberta Ministry of Health. We used the Alberta Health Care Insurance Plan Central Stakeholder Registry (AHCIP/CSR) to identify deaths, departures from the province and location of residence (postal codes). Using data on hospital admissions (Hospital Morbidity Inpatient Database) and physician claims (Fee-for-Service Administrative Claims Database), we identified incident cases of HZ between 1993 and 2018. Case data from outpatient and emergency department visits (Ambulatory Care Classification System) was included for 1997–2018. Prior to 1997, data on outpatient and emergency department visits was captured in the Fee-for-Service Administrative Claims Database. We obtained vaccination status from the provincial Immunisation and Adverse Reactions to Immunisation database (Imm/ARI), which contains individual-level vaccination information for publicly funded vaccines. We excluded First Nations status children, individuals born in Lloydminster and those born in Edmonton prior to 2005 from the analysis, as vaccination data for these populations are not captured in Alberta’s provincial database.

### Case definition

We defined a case of medically attended HZ as an individual with an ICD-9 code of 053 or ICD-10-CA code of B02 in physician, outpatient or hospital inpatient records. Only incident cases, defined as the first HZ-associated outpatient visit or hospitalisation during the study period for each individual, were included. These HZ diagnostic codes have relatively high positive predictive values (PPV) of 87% [12] and 97% [5] for children aged 0–17 years.

### Data analysis

To calculate HZ cumulative incidence rates for each age group, we used person-years at risk. Each individual contributed person-time at risk on entry into the study cohort, either at date of insurance activation (date of birth) or start of study period. The individual stopped contributing person-time upon exiting the cohort (through death, relocation or age), contracting HZ or at the end of the study period. Subjects could contribute both vaccinated and unvaccinated person-time to the study.

We calculated cumulative incidence rates for four unique populations, defined by vaccine availability period (pre-universal vaccination era [birth cohort 1993–1999], universal vaccination era [birth cohort 2000–2018]) and vaccination status (vaccinated/unvaccinated). We estimated age-specific incidence rates for the following: (1) vaccinated in pre-universal vaccination era, (2) unvaccinated in pre-universal vaccination era, (3) vaccinated in universal vaccination era and (4) unvaccinated in universal vaccination era. To evaluate the impact of vaccination status on HZ risk, we calculated incidence rate ratios (IRRs) by comparing the HZ incidence in those vaccinated compared to unvaccinated, in both the pre-universal and universal vaccination eras. To evaluate the impact of vaccine availability period, we calculated IRRs by comparing the incidence rates in pre-universal and universal eras, in both the vaccinated and unvaccinated populations. In both cases, we estimated the total IRR and the IRRs by age group.

To evaluate the impact of the two-dose programme on HZ risk, we compared cumulative HZ incidence rates after two doses of vaccine to incidence rates after one dose. Only children eligible for the two-dose programme (i.e. birth cohort 2006–2018) were included in this analysis. For one-dose incidence rates, person-years at risk began after receipt of first vaccine dose and ended with exiting the cohort (through death, relocation or age), contracting HZ, completion of study period or receipt of second dose of vaccine. Two-dose time at risk began on receipt of second dose of vaccine and ended the same as one-dose time at risk. Subjects could contribute person-time to both one-dose and two-dose incidence rates.

Annual HZ incidence rates were calculated for each age group for the entire study period (1993–2018), by dividing the total number of HZ cases in the year in Alberta by the mid-year population estimates from provincial vital statistics data. Incident cases were defined as cases with no HZ diagnosis within the previous 180 days. Annual incidence rates were expressed as cases per 100,000 person-years.

We calculated cumulative and annual HZ incidence rates using SAS v9.4 and IRRs and 95% confidence intervals using OpenEpiv3.01.

## Results

After excluding children born in Lloydminster (n = 7,854), born before 2005 in Edmonton (n = 124,552) or identified as First Nations status (n = 74,562), the final study cohort contained 924,124 children aged < 20 years, contributing a total of 9,257,601 person-years of follow-up time. There was a total of 11,622 incident HZ cases in the study population during the study period (Table 1). Prior to universal vaccination, HZ cases were distributed relatively evenly across all age groups greater than 1 year of age, with the number of cases slightly higher in older age groups. After the universal vaccination programme was implemented, almost 50% of cases occurred among those less than 5 years of age.

**Table 1:** Distribution of herpes zoster cases by varicella vaccine availability, 1993–2018

Variable	Pre-universal vaccination era (1993–1999)		Universal vaccination era (2000–2018)	
	Number of HZ cases/study population	Percentage	Number of HZ cases/study population	Percentage
Sex				
Female	3638/87,836	4.1	2417/366,636	0.7
Male	2984/83,295	3.6	2583/386,353	0.7
Vaccination				
Yes	542/48,787	1.1	3509/670,939	0.5
No	6080/122,345	5.0	1491/82,053	1.8
	Number of HZ cases	Percentage (n = 6,622)	Number of HZ cases	Percentage (n = 5,000)
Age range (years)				
< 1	242	3.6	437	8.7
1–4	1365	20.6	1992	39.8
5–9	1596	24.1	1375	27.5
10–14	1621	24.5	872	17.4
15–19	1798	27.1	324	6.5
Vaccine doses^a^				
1 dose	NA	NA	1553	76.4
2 doses	NA	NA	480	23.6

*HZ*, herpes zoster; *NA*, not applicable

^a^HZ cases only include those eligible for the two-dose programme (birth cohort 2006–2018), n = 2,033

**Fig. 1:**
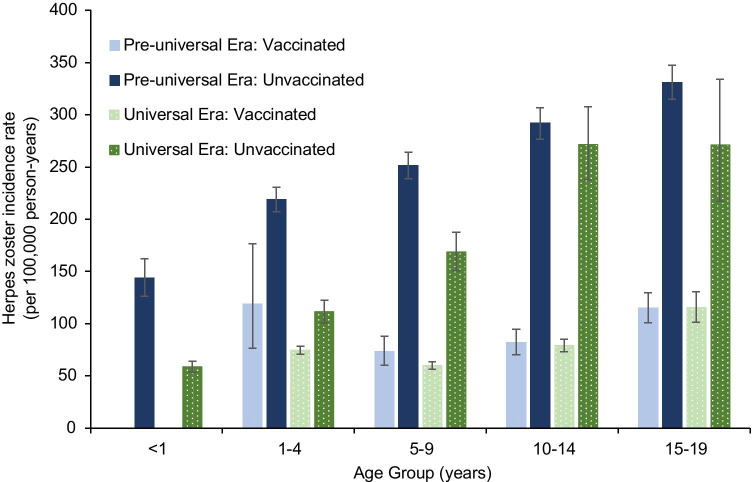
Age-specific herpes zoster incidence rates by vaccine availability period and individual vaccination status. Bars show 95% confidence intervals for the point estimates

When stratified by vaccination era, rates of HZ were significantly higher in the unvaccinated population compared to the vaccinated population across all age groups (Fig. 1). The overall HZ rate among unvaccinated children was 259.4 (95% CI: 253.0, 266.0) and 104.4 (95% CI: 99.2, 109.8) per 100,000 person-years in the pre-universal and universal eras, respectively. During these two time periods, HZ rates in vaccinated children were 92.8 (95% CI: 85.3, 100.0) and 71.6 (95% CI: 69.2, 74.0) per 100,000 person-years. HZ incidence increased in each subsequent age group among unvaccinated children born prior to universal vaccination (Fig. 1). This effect was also noted for the unvaccinated cohort, who were eligible for universal vaccination, though the difference between the oldest two age groups was not significant (Fig. 1).

HZ incidence was 64% higher in the unvaccinated group than the vaccinated group during the pre-universal vaccination era (IRR: 0.36, 95% CI: 0.33, 0.39), and 32% higher during the universal vaccination era (IRR: 0.68, 95% CI: 0.64, 0.73) (Table 2). Vaccination was consistently related to decreased HZ risk across all eligible age groups during both eras. Among unvaccinated groups, HZ rates were lower among those born during the universal vaccination period than those born before universal vaccination; however, this difference was not statistically significant for children older than 9 years of age (Table 2). For vaccinated children, the overall HZ risk was also lower during the universal vaccination era compared to the pre-universal vaccination era; however, when evaluated by age group, this finding was only significant for 1–4-year-olds (Table 2).

**Table 2:** Age-specific estimated risk ratio of herpes zoster incidence by vaccination status, vaccine availability period and number of doses

Age range (years)	Risk of HZ, IRR (95% CI)				
	Vaccination status, vaccinated vs. unvaccinated		Vaccine availability period, universal vaccination era (2000–2018) vs. pre-universal vaccination era (1993–1999)		Number of vaccine doses, 2 doses vs. 1 dose (2006–2018)
	Pre-universal era (1993–1999)	Universal vaccination era (2000–2018)	Vaccinated	Unvaccinated	
< 1	NA^a^	NA^a^	NA^a^	0.41 (0.35–0.48)	NA^a^
1–4	0.54 (0.36–0.83)	0.67 (0.60–0.74)	0.63 (0.41–0.96)	0.51 (0.46–0.57)	0.52 (0.38–0.73)
5–9	0.29 (0.24–0.35)	0.35 (0.31–0.40)	0.82 (0.67–1.0)	0.67 (0.59–0.76)	0.63 (0.54–0.73)
10–14	0.28 (0.24–0.33)	0.29 (0.25–0.34)	0.97 (0.82–1.15)	0.93 (0.81–1.07)	0.52 (0.39–0.68)
15–19	0.35 (0.31–0.40)	0.42 (0.33–0.55)	1.00 (0.84–1.2)	0.82 (0.66–1.02)	NA^b^
Total^c^	0.36 (0.33–0.39)	0.68 (0.64–0.73)	0.77 (0.70–0.84)	0.40 (0.38–0.43)^d^	0.59 (0.53–0.65)^e^

*CI*, confidence intervals; *HZ*, herpes zoster; *IRR*, incidence rate ratio; *NA*, not applicable

^a^Unable to calculate IRR for vaccinated state, as no subjects in this age group had a history of vaccination

^b^Subjects in this age group not eligible for universal two-dose vaccination during study period

^c^IRR calculated for age group 1–19 years unless otherwise specified

^d^IRR calculated for 0–19 years

^e^IRR calculated for 1–14 years

**Fig. 2:**
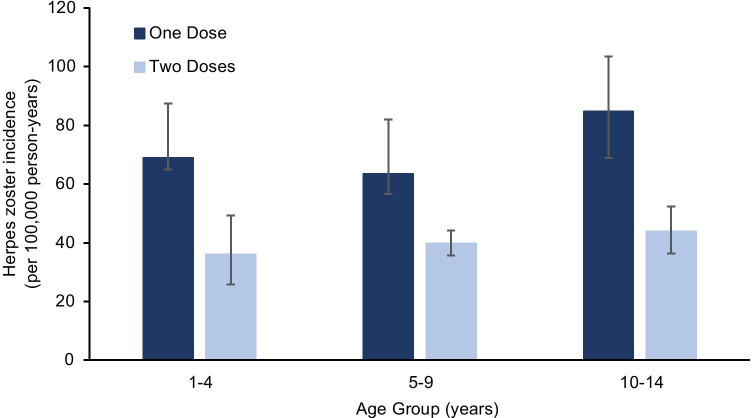
Age-specific herpes zoster incidence rates by number of vaccine doses received, birth cohort 2006–2018. Bars show 95% confidence intervals for the point estimates

Among children born after 2005, HZ incidence rates were significantly lower after receipt of two doses of vaccine, compared to rates after only one dose (Fig. 2). Children who received two doses of vaccine (40.3 HZ cases per 100,000 person-years) had approximately 41% lower risk of HZ (IRR: 0.59, 95% CI: 0.53, 0.65) than those who received only one dose of vaccine (68.4 HZ cases per 100,000 person-years). This decreased risk was similar across all eligible age groups, ranging from 37% for the 5–9 years age group to 48% for the 10–14 and 1–4 years age groups (Fig. 2; Table 2)*.*

**Fig. 3:**
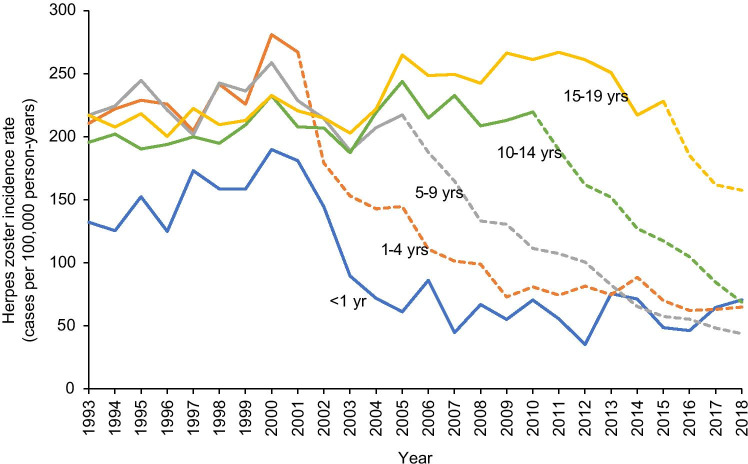
Annual herpes zoster incidence rates, by age. Rates include both vaccinated and unvaccinated subjects. Dashed lines represent annual herpes zoster incidence rates during universal vaccination eligibility. For each age group, the dashed line begins when subjects eligible for universal vaccination (i.e. birth cohort 2000–2018) are old enough to be included in the group

The annual incidence of paediatric HZ cases remained between 205 and 246 HZ cases per 100,000 years in 1993–2001 and declined ~ 64% after implementation of the universal vaccination programme, from 226 (2001) to 82 HZ cases per 100,000 person-years (2018). We noted a similar pattern among all age groups, though the decrease began progressively later with increasing age, generally corresponding to the initiation of the routine vaccination programme for each age cohort (Fig. 3). Incidences declined by 70–80% among all age groups except the oldest (15–19 years), which declined 41%, from 267 to 158 HZ cases per 100,000 person-years between 2011 and 2018.

## Discussion

This study expands upon prior work evaluating the impact of varicella vaccination on paediatric HZ risk by including individual-level vaccination data for a population with universal publicly funded health care. Our study incorporates 25 years of data, including 18 years of a universal, publicly funded vaccination programme.

As expected, varicella vaccination was consistent with reduced HZ risk in children across all age groups, corresponding to decreased HZ risk of 64% and 32% in the pre-universal vaccination and universal vaccination eras, respectively. The decreased impact of vaccination during the universal vaccination era is largely due to the decrease in HZ incidence among unvaccinated children observed after implementation of the universal vaccination programme. Notably, this impact was significant even among those less than 1 year of age, who are not eligible for varicella vaccination. The decreased risk among unvaccinated groups in the universal vaccination era, during which vaccine coverage was > 80% [1, 13], compared to the pre-universal vaccination era, with vaccine coverage < 5% [1], indicates that herd immunity can have a significant impact on HZ risk. Although the decreased risk was non-significant for the two oldest age groups, these age groups reflect the early years of the universal vaccination programme, when the likelihood of exposure to wt-VZV (and thus HZ risk) was higher. Literature consistently reports reduced HZ risk among vaccinated versus unvaccinated children, with reported values for HZ rates during universal vaccination programmes ranging from 66 to 92% lower in vaccinated compared to unvaccinated populations [3–6]. This reduction in paediatric shingles incidence is comparable with what we observed during the pre-universal vaccination era (64%) but is higher than what we found during the universal vaccination programme era (32%). These studies were conducted earlier in universal vaccination eras [3, 5, 6] or included children born prior to universal vaccination programmes [4]. Thus, findings may reflect lower vaccination coverage in less mature vaccine programmes, with the effects of herd immunity not yet impacting HZ rates among unvaccinated, which we posit ultimately decreases the difference in HZ risk between vaccinated and unvaccinated children.

Our results also demonstrate that the overall HZ incidence rate among vaccinated children was lower in the universal vaccination period than the pre-universal vaccination era, suggesting HZ risk among vaccinated children will decrease as circulating wt-VZV decreases. Similarly, Weinmann et al. (2013) found approximately half of HZ cases among vaccinated subjects was associated with wt-VZV [5]. However, without laboratory confirmation, we cannot determine whether the proportion of HZ incidence due to wt-VZV has changed over time.

The HZ rates we determined for vaccinated children during both time periods (92.8 and 71.6 HZ cases per 100,000 person-years during the pre-universal and universal eras, respectively) were consistently higher than rates reported in the literature (17.9–48 HZ cases per 100,000 person-years) [3–5, 14]. Published rates are all from studies conducted in the USA, so may reflect different patterns in health care access. Differences in vaccines employed may also contribute to differences in baseline HZ risks among vaccinated children, though these differences are likely small [14]. Methodological differences may have contributed to differences in results, as we were unable to verify results as with prospective studies [3, 5] and used a less restrictive definition of HZ than the other administrative data studies. Previous studies using administrative data have excluded postherpetic neuralgia (PHN)-specific codes, on the argument that these diagnoses might not represent incident HZ cases [4, 14]. However, Marra et al. [2] found that PHN-specific codes excluded only about 0.05% of HZ cases in overall population (age 0–80 +).

Among children who received two doses of vaccine, HZ rates were approximately 41% lower than those who received only one dose of vaccine. This difference was noted even in the 1–4 age group, of which only 4-year-olds would have been eligible for the second dose. This may be a reflection of higher HZ risk in the first year after one-dose vaccination [4], rather than a difference between one- and two-dose recipients. The decreased HZ risk among two-dose vs. one-dose recipients is similar to results reported elsewhere (36% [14] and 50% [4]).

The consistent level of HZ risk among vaccinated children during the universal vaccination era indicates that protective effects of vaccination persist throughout childhood and adolescence, as most children would have received first-dose vaccination before age two. HZ risk among the vaccinated 15–19 age group is higher than the other vaccinated age groups; however, this may be a reflection of the impact of two-dose vaccination, as this age group would not have been eligible for a second vaccine dose. In contrast, among unvaccinated subjects, increasing age does appear to be related to increasing HZ incidence. Using administrative data from across the USA, Harpaz and Leung [15] also observed a positive relationship between HZ incidence and age among children under 17 years of age, though they found this relationship was no longer apparent in the cohort eligible for universal varicella vaccination. However, this study did not differentiate between vaccinated and unvaccinated subjects, which may have masked the potential influence of age on HZ risk among unvaccinated children. Further follow-up is required to determine whether the observed relationship between age and HZ risk will be muted as the universal vaccination programme matures, and children with minimal exposure to wt-VZV age. In addition, it will be important to clarify whether this relationship is an artifact of other confirmed risk factors such as immunocompromised status.

This is the first Canadian study to find a decrease in annual HZ incidence for children > 10 years of age after implementation of universal vaccination; however, this is likely due to the shorter follow-up time of previous studies [1, 2, 16]. Results are consistent with recent studies in the USA examining paediatric HZ incidence for a similar length of time after initiation of universal vaccination programmes [4, 15, 17]. The progressively later declines with increasing age found here and elsewhere [15] support the idea that population-level decreased incidence will occur as cohorts with high vaccine coverage, and thus lower risk of exposure to wt-VZV, age.

### Limitations

We were unable to determine history of wt-VZV exposure, as varicella infections often do not result in medical care [18] and are not reliably reported through infectious disease surveillance [19]. Privately purchased vaccines were not included in our analysis, though the short period of availability and low coverage rates during that time period indicates few children were vaccinated privately. We were unable to include First Nations children in our study. Given potential disparities in vaccine coverage [20] and varicella/HZ incidence in this population [21], this is an important area for future research. Immunocompromised children were not differentiated in the analyses. Immunosuppression is a significant risk factor for HZ [22] and may also be a contradiction for varicella vaccination; therefore, reported rates likely overestimate HZ incidence among healthy children, particularly those who remain unvaccinated. We did not verify the diagnostic codes used in our analysis. Researchers have reported high PPV for these codes [5, 12]; however, HZ diagnosis misclassification may overestimate the number of true HZ cases, particularly among younger children [6, 12]. In addition, unvaccinated children may be more likely to be diagnosed with HZ, potentially due to differences in patient presentation [3] or physician awareness [5]. To date, none of the studies evaluating HZ epidemiology in Canada have validated HZ diagnostic codes [10]. These factors highlight the need for on-going validation studies for paediatric HZ.

## Conclusions

Paediatric HZ incidence rates in Alberta significantly decreased after implementation of a universal varicella vaccination programme. The risk of paediatric HZ has decreased in all age groups, in both vaccinated and unvaccinated individuals. The indirect benefits of the programme are crucially important for maintaining the safety of immunocompromised children and children < 1 year of age, as they may be ineligible for vaccination and at higher risk for HZ incidence and associated complications.

This analysis helps to clarify the impact of a universal varicella vaccination programme on paediatric HZ incidence, which can provide insight for national advisory committees on immunisation when considering implementation of a varicella vaccination programme. Continued monitoring of HZ incidence in countries with universal varicella vaccination programmes is required to further assess the longevity of vaccine-associated protection.

## Data Availability

The data was collected as part of the routine collection of administrative data by the Alberta Ministry of Health. Access requests must be submitted to the Alberta Ministry of Health.
